# Food Security Characteristics Vary for Undergraduate and Graduate Students at a Midwest University

**DOI:** 10.3390/ijerph18115730

**Published:** 2021-05-26

**Authors:** Molly B. Hiller, Donna M. Winham, Simon T. Knoblauch, Mack C. Shelley

**Affiliations:** 1 Department of Food Science & Human Nutrition, Iowa State University, Ames, IA 50010, USA; molhiller@gmail.com (M.B.H.); simonk@iastate.edu (S.T.K.); 2 Department of Political Science, and Department of Statistics, Iowa State University, Ames, IA 50010, USA; mshelley@iastate.edu

**Keywords:** food insecurity, young adults, college students, socioecological model, food environment

## Abstract

The study objective was to determine prevalence of food insecurity and its associations with socioecological model (SEM) characteristics for undergraduate and graduate students. An online questionnaire was distributed to a convenience sample of students aged 18–34 at a Midwestern university. Of the 938 responses, 675 were complete for analysis. Outcome measures included demographics, food security level, housing, food access barriers, coping strategies, and food assistance program usage. Results found that predictors associated with undergraduate food insecurity included non-White race, receipt of financial aid, lower self-reported health status, living off-campus, employment, and food cost (*p* < 0.001). Graduate student food insecurity was associated with Asian self-identification, employment, food cost, no time to prepare foods, and lack of foods for dietary needs (*p* < 0.001). Students with food insecurity were more likely to buy cheap food (*p* < 0.001). Almost 50% of food-insecure undergraduates asked friends or family to help buy food. Food-insecure students were more likely to want information on meal preparation and budgeting. More graduate students were likely to know of and use food pantries. Overall, food insecurity was higher among undergraduate than graduate students. Universities should consider institutional and policy changes tailored to the separate populations to mitigate the prevalence of campus food insecurity.

## 1. Introduction

The high prevalence of food insecurity among college students has received increasing attention in the past decade. Food insecurity is defined as the lack of financial, physical, and social means to obtain safe and nutritious foods meeting one’s preferences and needs for a healthy lifestyle [1]. Identifying the factors affecting food insecurity is necessary to ameliorate the condition through improved aid policies and other forms of assistance. This is essential to ensuring the health and academic success of college students. Such identification of factors related to food insecurity need to account for differences between major subgroups of students. For example, studies indicate that, due to differences in living and social situations, cooking expertise, and finances, predictors of food insecurity may vary between undergraduate and graduate students [2,3,4]. However, much is not known about how each group experiences food insecurity. This study aims to develop separate predictive models of food insecurity status for undergraduate and graduate college student populations that account for multiple factors and their interactions and will support the future design of interventions and messaging that will be effective in reducing the problem.

Prior studies have examined food insecurity frequency in college students. One systematic review found that 14–33% of students were food insecure during their academic career, compared to 11% of the general population [5,6]. At an Oregon university, 59% of students reported food insecurity within the previous year, and other university students indicated significantly higher rates of food insecurity at the end of a semester compared to the beginning of the school year [7,8]. These differences highlight the fluctuating and situational nature of the problem and suggest students may not have enough aid or other financial support to last an entire school year.

Recognition of the high levels of food insecurity on college campuses has led to increased organizational efforts and changes in institutional policies offering food pantries and outreach to students [9]. Government legislation has been introduced to address the issue of college food insecurity, although it remains to be seen how successful or effective their implementation will be, especially in light of the coronavirus pandemic [10].

While earlier investigations examined the relationship between food security and various population characteristics, few examined the inherently different barriers that undergraduate and graduate students may face regarding food access. A 2019 study found food security factors common to all students (race/ethnicity, perceived health rating, financial aid, employment status) as well as other patterns that were specific to each group [2]. Some studies have found that undergraduates are more food insecure than graduate students [2,3]. Graduate students have different characteristics: they are generally older, may be partnered, possibly have dependents, and are more likely to live off-campus and be employed. They also have greater nutrition knowledge and cooking skill sets than younger, more frequently single, and on-campus undergraduates. International students are generally more represented among graduate students. They may face challenges accessing culturally desirable food and have external work permit or income restrictions [2].

**Figure 1:**
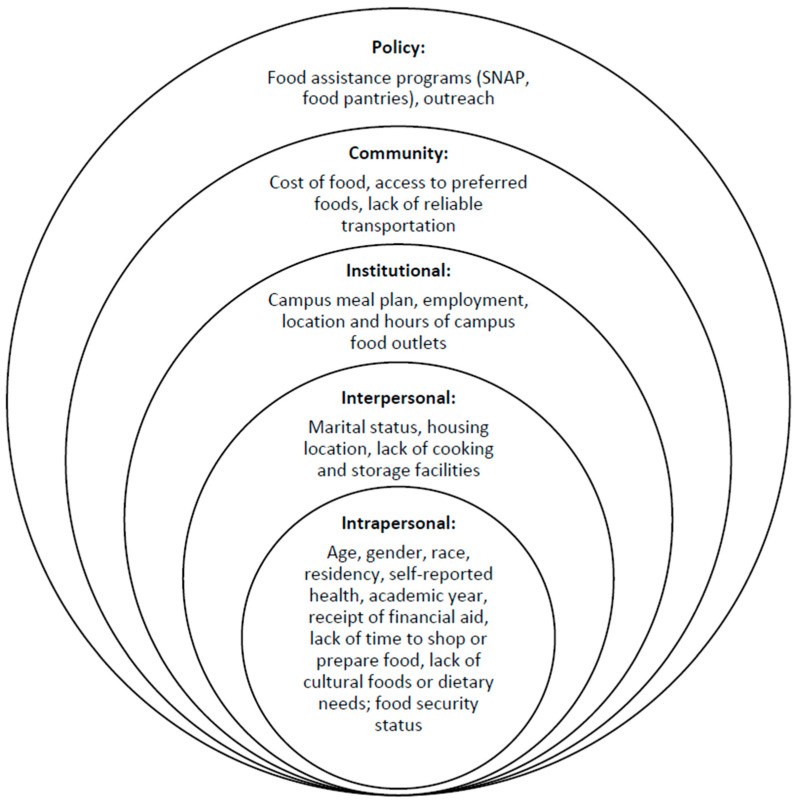
Socioecological model (SEM) levels of influence likely to affect college food insecurity.

Studies of food insecurity indicate that the condition is influenced by multiple interactions between social, behavioral, and environmental factors that can be categorized into five levels: intrapersonal, interpersonal, institutional, community, and policy [11,12]. Such levels are embodied in the socioecological model (SEM) framework (Figure 1), which we adopt as a basis of our predictive models. The SEM provides a framework for examining barriers that go beyond personal finances and can inform the development of strategies for dealing with food insecurity and interventions that would have the most likelihood of success.

As modeled by the first level of the SEM, intrapersonal characteristics such as age, gender, race, culture, academic year, income, and health status are related to food insecurity. Some studies have suggested that men are more likely to be food insecure than women [2,13]. Students of color, first-generation students, and single parents with dependent children are also more likely to face food insecurity [5,13,14]. Individuals become vulnerable after transitioning to a higher learning setting due to decreased food access overall (e.g., colleges located in food deserts) and limited availability of desired or culturally acceptable foods [13,15]. Though likely multifactorial, one contributing factor to this increased risk may be college students’ newfound independence and having limited confidence in their financial management skills [13]. Financial aid covers college expenses but does not necessarily provide cash for living expenses such as food purchases. Food-insecure students are more likely to self-report their health as “poor” or “fair” as well as report adverse mental health conditions such as depression and anxiety [7,16,17].

On the interpersonal level, factors such as housing situation (e.g., on- or off-campus, single or with partner) reflect social interactions. The changing social support during college may influence food security in positive or negative ways. Undergraduate students are often away from home for the first time and may be uprooted from familiar meal patterns, food sources, cultural practices, and regular schedules [18]. New peer groups and social circumstances can affect food decisions [15,19].

At the institutional level, aspects of the college environment such as inadequate campus meal plans, campus food outlets, and food preparation facilities as part of housing can contribute to food insecurity [3,15,20]. Campus meal plans are relatively expensive yet provide accessible prepared foods, though their offerings may vary in cultural acceptability among minorities such as international students. Campus residence halls may not have facilities for food preparation. Tuition, housing, and other financial obligations may compete with nutritional needs [20,21].

The community environment near colleges further influences food accessibility. Lack of public transportation may limit access to retail food outlets and restaurants [3,17,21]. While food assistance programs or facilities such as the national Supplemental Nutrition Assistance Program (SNAP) or local food pantries are often available, students do not always know of these resources or face eligibility restrictions [22].

In the present study, the SEM was adapted to guide data collection and analysis by reflecting the unique circumstances of college students and food security by undergraduate and graduate student status, with the goal of identifying the most important predictive factors for the separate groups. A cross-sectional survey was conducted to determine the prevalence of food insecurity and to determine associations with SEM characteristics and food insecurity for undergraduate and graduate students. It was hypothesized that (1) undergraduates would have different levels of food insecurity than graduate students, and (2) SEM variables associated with food insecurity would be different for undergraduate and graduate students.

## 2. Materials and Methods

### 2.1. Study Participants

The overarching study purpose was to examine dietary acculturation and ethnic identity affiliation among international students, in addition to describing food security characteristics (data reported elsewhere). Therefore, this pilot study included two of seven colleges at Iowa State University based on their level of ethnic diversity and size of international students. Enrolled students in the College of Business (*n* = 4921) and the College of Liberal Arts and Sciences (*n* = 8284) received a direct email survey invitation in April 2018. Students age 18–34 years were eligible. Participants who completed the survey received a $5 gift card to a major retailer as an incentive. The Iowa State University Institutional Review Board deemed this study exempt (#16-289).

### 2.2. Survey Instrument

The survey questions were structured to reflect SEM levels of influence likely to affect food security (Figure 1) based on the literature review. The intrapersonal SEM characteristics included age, gender, race, residency status (in-state, out-of-state, international), self-reported health status, academic year, and receipt of need-based financial aid as a proxy for income [23,24,25]. Other intrapersonal barriers to food access were lack of time to shop or prepare food, limited availability of cultural foods, or specific dietary needs with a reference time frame of the current semester [23]. Food security status as estimated by the USDA 10-Question Adult Food Security Module over the past 12 months was considered an intrapersonal factor [25].

Interpersonal characteristics were marital status and housing location (on-campus or with parents, off-campus), and the lack of facilities to cook or store food [23,24]. Institutional influences included the use of a campus meal plan, employment, and the location and hours of operation of campus food outlets [24]. SEM community-level characteristics included the food environment and availability of items, cost of food, access to preferred cultural foods, and lack of reliable transportation [23,24]. Awareness and use of food assistance programs, including SNAP and local food pantries, were considered the policy level of influence [23,26].

### 2.3. Data Analysis and Transformations

Online responses from Survey Monkey (Palo Alto, CA, USA) were downloaded into SPSS Version 26.0 (IBM, Armonk, NY, USA). Food security raw scores, ranging from 0 to 10 points, were categorized into four levels of food security (high, marginal, low, very low) for reporting and food secure/food insecure for analysis, following USDA module guidelines [25]. Variables were examined for normality of distribution. Ordinal variables with small cell counts were condensed for table display (e.g., self-reported health status, frequencies for coping strategies). Categorical variables were compared by undergraduate/graduate student status, and within these groups by food secure/insecure status using chi-square tests for independence. Variables were also compared by gender, residency (domestic/international), and housing location (on-campus/off-campus) as checks for possible group differences or confounders (data not shown). Logistic regression modeling was applied to predict food security status for graduate and undergraduate students separately. Bivariate variables were recoded as dummy variables for regression analysis with food insecurity, female gender, non-White race, and receipt of financial aid as reference categories. *p* values <0.05 were considered significant.

## 3. Results

Among the 14,841 students who received a direct email invitation, 6750 opened the link, and 938 started the survey. Thirteen did not answer any questions, 17 were ineligible due to age or college enrollment, and 233 were incomplete for the variables of interest. Demographic characteristics are shown in Table 1. Most of the 675 participants were undergraduates (81%), female (66%), non-Hispanic White (79%), and in-state residents (60%). Just over 2% reported being a parent, and less than 7% self-identified as Hispanic (data not shown). In comparison, Iowa State University’s overall 2018 demographics for the 34,992 students enrolled were 85% undergraduates, 71% non-Hispanic White, and 59% in-state residents. Fewer than 6% were Hispanic and 43% were female [27].

Compared to undergraduates, significantly more graduate students were Asian, international, married or cohabitating, living off-campus, employed on-campus, and had higher incomes. Undergraduates were more likely to have a campus meal plan and work off-campus. Sixty-eight percent of the total sample was food secure (47.1% high; 21.1% marginal) and 32% was food insecure (16.3% low; 15.5% very low).

For undergraduates, food security status did not differ by intrapersonal factors of gender, residency status, or marital status. Significantly higher proportions of Asian, Black, or biracial students were food insecure compared to Whites. More of the food-insecure undergraduates received need-based financial aid. For interpersonal characteristics, undergraduate students living in off-campus housing were significantly more likely to be food insecure than those in campus residence halls or living with parents. Undergraduates who were not working were more food secure than those who were currently employed.

Unlike undergraduates, most demographic traits were not significantly different by food security status for graduate students. Self-reported health was rated better by food-secure students than their food-insecure peers for both undergraduate and graduate students. At the community level, those who were food insecure utilized food pantries significantly more than those who were food secure among both undergraduate and graduate students.

Table 2 shows the frequency distributions by food security/insecurity for undergraduate and graduate students regarding barriers to food access. Over 50% of the food-insecure in both cohorts reported they very often/often had no time to prepare food, with 40% saying the same for food shopping. All nine barriers to food access significantly affected food-insecure undergraduates more than those who were food secure. For graduate students, those who were food insecure had more limitations on facilities to prepare food, location of campus food outlets, costs of food, and reliable transportation than their food-secure peers.

Table 3 shows the coping strategies for financial decisions: buying the cheapest food available, asking family or friends to cover food costs, having to choose between using money for food or for medical care, and using a food pantry. All were significantly different for food-secure versus food-insecure students at both undergraduate and graduate levels, with food-insecure students using food security coping responses more often. The most frequent coping strategy was purchasing the cheapest food despite knowing it was not healthy. Almost 40% of food-insecure undergraduates and 19% of food-insecure graduates reported using this food security strategy every month. In contrast, approximately three times as many food-insecure graduate students used food pantries than undergraduates.

Student knowledge of, interest in using, and usage of campus and community resources for meal preparation, budgeting, food assistance, food pantries, and nutrition assistance programs are shown in Table 4. Expressed need for information on meal preparation (38%) and budgeting (32%) was relatively high. Markedly fewer respondents were interested in information on campus resources if having difficulty with obtaining food (15%), location of food pantries (14%), or federal food assistance programs (11%). For undergraduates, all of the five resource and program usage responses were significantly different by food security status. Approximately 45% of the food-insecure cohort wanted information on meal preparation and budgeting, with 25% interested in campus resources and the location of food pantries. Graduate and undergraduate students showed similar patterns for resources on meal preparation, budgeting, and campus resources. Thirty-seven percent of food-insecure graduate students wanted to know how to apply for federal nutrition assistance such as SNAP.

A logistic regression model was estimated to ascertain whether SEM variables were associated with food insecurity status for graduate and undergraduate students separately. Seven intrapersonal variables were used in both models (age, gender, race, self-reported health, no time to prepare food, no time to shop for food, lack of foods for dietary needs). Academic year and receipt of financial aid were in the undergraduate model and residency (in-state, out-of-state, international) was added to the graduate student model. Three interpersonal factors (marital status, housing location, lack of facilities to cook or store foods), four institutional traits (use of campus meal plan, employment, campus food outlet locations, and hours of operation as barriers), and three community characteristics (cost of food, lack of cultural foods, lack of reliable transportation) were in both models.

Table 5 shows the most parsimonious significant model (*p* < 0.001) for undergraduates, which included six predictors (intrapersonal: self-reported health, receipt of financial aid; interpersonal: housing location, employment; institutional: campus food location hours; community: cost of food). The model correctly classified 80.6% of food-security outcomes, including 87.3% instances of food security and 67.7% of food insecurity. Logistic regression fit metrics indicate that the model performs adequately (Cox and Snell pseudo-*R*^2^ = 0.376 and Nagelkerke pseudo-*R*^2^ = 0.520). Undergraduates who perceived the cost of food as a barrier were three times more likely to be food insecure (OR 3.00). Those who received financial aid (OR 2.77), and those who lived off campus (OR 2.45) were more than twice as likely to be food insecure. Students with lower self-reported views of health were 1.8 times more likely to be food insecure. The graduate student final model included five predictors (intrapersonal: Asian/other race, lack of foods for dietary needs, no time to prepare food; interpersonal: employment; community: cost of food). The model correctly classified 89.5% of food-security outcomes, including 95.9% instances of food security and 66.7% of food insecurity. Graduate students of Asian descent were 5.7 times more likely to be food insecure than their peers. Those who stated the cost of food was often a barrier were more likely to be food insecure (OR 7.8). Logistic regression fit metrics indicate that the graduate student model performs adequately (Cox and Snell pseudo-*R*^2^ = 0.397 and Nagelkerke pseudo-*R*^2^ = 0.611). The Hosmer–Lemeshow goodness-of-fit test was not significant for either undergraduates or graduates, which indicates that the models are well calibrated to understand the influences underlying food insecurity and thus provide a strong foundation for recommendations for both policy and practice.

## 4. Discussion

The study objective was to investigate the prevalence of food insecurity by undergraduate and graduate student status and to investigate associated factors using a SEM approach. It was predicted that undergraduates would have different levels of food insecure than graduate students, and that SEM variables associated with food insecurity would differ between the cohorts. Findings identified the prevalence and confirmed the hypothesis that food security differed by cohort, with undergraduates (34%) more likely than graduate students (21%) to be food insecure. This result aligns with the few other studies that have compared the two academic groups [2,3]. Results also identified characteristics of the SEM associated with food insecurity overall or that differed by undergraduate and graduate student status. Recognizing these nuances may help universities improve their practices and messaging to best serve a diverse student body and increase food security.

Intrapersonal SEM variables related to food insecurity varied by undergraduate and graduate cohorts. Non-Hispanic White undergraduate students had higher proportions of food security than Asian and Black or other students, and Asian graduate students appeared at substantially higher risk of food insecurity. Other studies have noted race/ethnicity as an intrapersonal characteristic associated with food insecurity [7,26,28,29]. These results support the need for examining disaggregated data by more than a non-Hispanic White and Other dichotomy [30]. While the topic of college student food insecurity has received increased attention in recent years, it is often generalized and examined through an undergraduate viewpoint. The results of the current study highlight that certain subsets of the population (e.g., international Asian graduate students) are vulnerable to experiencing food insecurity. Opportunities to reach everyone affected by food insecurity on campuses may be missed if interventions designed to mitigate its prevalence do not consider these distinctions. The presence of appropriate, culturally tailored resources is vital in developing rapport and credibility among food-insecure minority students [31].

No gender differences were observed, in contrast to two studies in the southern U.S. where undergraduate males were more likely than females to experience food insecurity [2,16]. Food-secure students were more likely to self-report their health status as better than their food-insecure peers. This mirrors several other studies in which food-insecure students more often rated their health as poor or fair [15,16]. Although it is a subjective measure, this common thread may suggest a cumulative effect of the challenges faced by students that are brought on and/or exacerbated by food insecurity. Other studies have noted links between food insecurity and increases in mental health issues [4].

Approximately 75% of food-insecure undergraduates received need-based financial aid compared to approximately half of food-secure undergraduates. Financial independence has been linked to higher risk for food insecurity among college students [21]. One 2018 study surveying food-insecure students found that 38% felt increased financial aid by the university would help to increase their food access [16].

Interpersonal SEM characteristics were associated with respondents’ level of food security, particularly by housing circumstances among undergraduates. Students living in on-campus residence halls or with parents experienced higher rates of food security than students living off campus. Other studies have suggested that food insecurity increased among those living off-campus alone or with roommates [3,32]. These differences underscore the dynamic relationship between financially dependent and independent undergraduates as well as the social interaction implications. More scrutiny of the potential hazards of transitioning to college, when food insecurity can emerge for students who had not experienced it previously, may be helpful in prevention [13].

Use of campus meal plans, a SEM institutional factor, was not significantly different in the current study for undergraduates. Meal plans are required for on-campus residents at Iowa State University, but they vary in the number of meals provided. A student may therefore select a meal plan that is more affordable but does not adequately cover their total food needs (e.g., a plan providing two meals daily instead of three). Although these results contrast with findings that meal plans were associated with higher food security among undergraduates, they suggest the need for ensuring that all students with meal plans have adequate access to food that fits their dietary needs and is desired and culturally appropriate [2]. Another study found that 43% of meal plan enrollees reported food insecurity [20].

Employment, a SEM institutional component, was significantly related to food insecurity. On-campus employment was more common than off-campus work. While seemingly paradoxical, employment has been commonly linked with food insecurity among students attending college [2,7]. This is likely due to the necessity of work to meet financial responsibilities, while unemployed undergraduates may have more financial support from family for living expenses. More details are needed on the nature of the employment.

Barriers to food access were spread across different components of the SEM. Intrapersonal concerns about lacking time to shop or prepare food were common among food-insecure undergraduates, as was the cost of food. The magnitude of lacking time for these tasks was about the same for all graduate students regardless of food security status. This suggests that for graduate students, lack of time to address dietary needs is connected with challenges beyond money alone. At the interpersonal level, lacking facilities to cook and store food was less of a problem for undergraduates but affected almost 20% of the food-insecure graduate students. A 2014 study found that higher food security was associated with greater perceived resource adequacy, such as time and adequate appliances for food preparation [21]. International students already facing obstacles in locating convenient or ready-to-eat culturally preferred foods may be further hampered if they lack the resources to prepare meals themselves.

An institutional barrier in the locations of campus stores was often an issue for both cohorts. Hours of operation were a concern for food-insecure undergraduates. This suggests that class or work hours interfere with the ability to purchase food at campus stores. Roughly one-third of food-insecure graduate students indicated that the location of campus food outlets was another barrier. Community-level concerns were evident, such as a lack of culturally appropriate foods for undergraduates and reliable transportation to obtain the food they desired for both groups. Limited transportation options have been cited by other studies as characteristic of food insecurity and/or preventing students from obtaining the types of food they want [16,33]. Similar to employment status, this likely reflects financial circumstances and one’s level of self-support.

The demographic and perceived barrier variables were used in the logistic regression model to evaluate associations of variables with food insecurity. Of the 12 predictors of food security among graduate and undergraduate students, six fell into the intrapersonal SEM sphere. These are largely immutable traits, in contrast with the one interpersonal, three institutional, and two community factors in the SEM that could be changed through policy. Common factors for both groups in the regression model were employment and cost of food as barriers.

Coping strategies for navigating food insecurity were seen across the levels of the SEM. Buying cheaper food that was less healthy was a relatively frequent practice, even for those who were not food insecure. Students may resolve themselves to less than healthy eating under the pressures of school and work, as it is a social norm to do so [34]. In both academic cohorts, the practice was high. Further research is needed to determine what those “cheap” foods are as defined by college students. Without knowing, it is problematic to suggest a food placement strategy for low-cost, healthier, and more accessible foods.

Asking friends or family for money was a more common practice among food-insecure undergraduates. The percentage of undergraduate and graduate students who spent their food money on social activities points to some of the conflicts faced by students when resources are scarce. Almost one-third of students had educational expenses that were prioritized over food at least some months of the year. While institutions may not be able to change cost policies, awareness of the potential magnitude of the problem may help them to target students at risk of attrition due to food insecurity and financial problems. Medical expenses prioritized over food was reported less frequently but still affected food-insecure students more. Greater use of food pantries by graduate students suggests they are savvier in finding resources, have better access to transportation to access sites, or experience less perceived stigma. They may also have dependents relying on them for food procurement.

There was high interest among all students in receiving information on cooking simple meals and budgeting at the intrapersonal level. Almost half of food-insecure students in both cohorts desired this information. Clearly, there is interest and need for guidance in this area. Institutional resources for food assistance were not as popular. More qualitative work on reasons why these services were less popular is needed. More food-insecure students were interested in community-level food pantry locations. In acknowledgment of the difficulties college students face with food access and eligibility barriers for most domestic nutrition assistance programs, universities have begun developing campus resources, including university-sponsored pantries [26]. A study looking at food pantry use identified barriers, including social stigma, self-identity, not having a clear understanding of food pantry policies, and inconvenient hours of operation [35].

At the policy level, food benefits from SNAP may be an underutilized resource for college students. A 2013 USDA report stated that roughly 25% of food-insecure college students received SNAP assistance [36,37]. However, students may not meet eligibility requirements for aid, such as working at least 20 h a week in addition to school or caring for a child [37]. A similar pattern was observed with more food-insecure graduate students wanting information about federal food assistance programs. While non-citizens are often ineligible for SNAP benefits, their dependents under age 18 can be, but may go without due to a lack of awareness and/or the perception of a hostile political climate. A study among low-income women in Arizona found that respondents native to the U.S. or those who were more acculturated were more likely to use SNAP despite similar levels of food insecurity [38]. With increased awareness and knowledge of available food assistance programs, universities may transform the campus food environment in a way that will positively affect food access for all students.

There are several limitations to this study. As a cross-sectional convenience sample, the results do not determine causation. Respondents from the two colleges included may not be representative of the university’s students overall or college students elsewhere. Data were self-reported and collected toward the end of the semester when monetary circumstances may have differed from other times during the academic year. Compared to general household food insecurity, college population research is relatively new and has no validated measurement tool. Prevalence rates are influenced by which measurement tools are utilized (e.g., the 6-item or 10-item USDA form, what timeframe respondents are being asked to reference) [39,40].

## 5. Conclusions

This research builds on evidence showing the widespread presence of food insecurity among the college student population, with approximately one-third of respondents designated as experiencing either low or very low food security. This study provides insights into the complex variables of food insecurity within a SEM framework by comparing undergraduate and graduate students. It is important to recognize these variables among the student population in attempts to improve food access for all those who struggle with it. While financial stressors play a major role in the problem and could be alleviated by improved aid policies and other forms of assistance, environmental circumstances should not be discounted. These include issues such as time, cooking skills, facilities to prepare food, campus outlet locations, the presence of culturally desirable offerings, and transportation for food procurement. The SEM looks at how factors beyond the individual are associated with behaviors, and this study shows that changing the environment may consequently influence behavior.

With increased awareness of the barriers students face, universities can take steps to recognize and mitigate the unique pitfalls of college life by engaging in multiple types of outreach. Future research may look at methods to improve food assistance program messaging to boost engagement and general awareness. Further, research could also address the self-efficacy and perceived health of students with food insecurity, who typically rate their health lower than their food-secure peers. Potential interventions with likelihood of acceptance and success could include time- and budget-friendly cooking classes, with guidance on how to cook inexpensive and easy, yet nutrient-dense meals, which would contribute to improving the overall health of college students. Universities may also consider campus planning efforts to increase access to adequate food sources.

## Figures and Tables

**Table 1:** Demographic characteristics of midwest university students by academic status ^1^.

Socioecological Model Characteristics	Total (*n* = 675) % (*n*)	Undergraduate 66% (*n* = 549)	Graduate 21% (*n* = 126)	*p*
INTRAPERSONAL				
Age in years ($\bar{x}$ ± SD)	21.7 ± 3.2	20.4 ± 1.9	27.7 ± 3.3	n.s.
Gender				n.s.
Male	33.6 (227)	33.0	36.5	
Female	66.4 (448)	67.0	63.5	
Ethnicity				
White	79.1 (534)	85.6 ^a^	50.8 ^b^	<0.001
Asian	16.5 (111)	10.6 ^a^	42.1 ^b^	
Black or Other	4.4 (30)	3.8 ^a^	7.1 ^a^	
Academic year				
Freshman	16.7 (113)	20.6		---
Sophomore	19.4 (131)	23.9		
Junior	21.8 (147)	26.7		
Senior	23.4 (158)	28.8		
Masters	5.3 (36)		28.6	
Doctorate	13.3 (90)		71.4	
Residency status				
In-state	59.9 (404)	68.1 ^a^	23.8 ^b^	<0.001
Out-of-state	20.8 (141)	22.8 ^a^	12.7 ^b^	
International	19.3 (130)	9.1 ^a^	63.5 ^b^	
Financial aid				
Yes	56.7 (383)	59.0 ^a^	46.8 ^b^	0.016
No	43.3 (292)	41.0 ^a^	53.2 ^b^	
Self-reported health				
Poor-Fair	15.7 (106)	16.8 ^a^	11.1 ^a^	n.s
Good	44.9 (303)	45.2 ^a^	43.7 ^b^	
Very Good	31.6 (213)	30.6 ^a^	35.7 ^b^	
Excellent	7.9 (53)	7.5 ^a^	9.5 ^a^	
INTERPERSONAL				
Marital status				
Single	88.0 (594)	93.1 ^a^	65.9 ^b^	<0.001
Married/Cohabit	12.0 (81)	6.9 ^a^	34.1 ^b^	
Housing location				
On campus or parents	31.9 (215)	38.4 ^a^	3.2 ^b^	<0.001
Off campus	68.1 (460)	61.6 ^a^	96.8 ^b^	
INSTITUTIONAL				
Campus meal plan				
No campus meals	65.7 (443)	59.9 ^a^	91.3 ^b^	<0.001
Campus meal plan	34.3 (231)	40.1 ^a^	8.7 ^b^	
Employment				
Not working	25.1 (169)	27.9 ^a^	12.8 ^b^	<0.001
On-campus job	51.3 (345)	43.6 ^a^	84.8 ^b^	
Off-campus job	23.6 (159)	28.5 ^a^	2.4 ^b^	

^1^ Statistical test used was chi-square analysis for independence; n.s. = non-significant. Same superscript letters indicate column proportions that are not significantly different from each other.

**Table 2:** Barriers to food access of midwest university students by academic and food security status ^1^.

		Undergraduate (*n* = 549)			Graduate (*n* = 126)		
Barriers to Food Access	Total (%)	Food Secure 66% (360)	Food Insecure 34% (189)	*p*	Food Secure 79% (99)	Food Insecure 21% (27)	*p*
INTRAPERSONAL							
No time to prepare food				<0.001			n.s.
Very Often–Often	42.3	34.4 ^a^	56.6 ^b^		41.4	51.9	
Sometimes	33.6	34.9 ^a^	31.2 ^b^		33.3	33.3	
Rarely–Never	24.1	30.7 ^a^	12.2 ^b^		25.3	14.8	
No time to shop for food				<0.001			n.s.
Very Often–Often	27.0	20.3 ^a^	40.7 ^b^		21.2	40.7	
Sometimes	35.0	32.6 ^a^	36.0 ^a^		42.4	33.3	
Rarely–Never	38.0	47.1 ^a^	23.3 ^b^		36.4	25.9	
Lack of foods for my dietary needs				<0.001			n.s.
Very Often–Often	9.6	5.8 ^a^	12.2 ^a^		15.2	22.2	
Sometimes	11.0	8.6 ^a^	14.8 ^b^		13.1	7.4	
Rarely–Never	79.4	85.5 ^a^	73.0 ^b^		71.7	70.4	
INTERPERSONAL							
Lack of facilities to cook or store food				0.045			0.011
Very Often–Often	16.3	17.0 ^a^	22.8 ^a^		2.0 ^a^	14.8 ^b^	
Sometimes	11.6	11.7 ^a^	16.4 ^a^		3.0 ^a^	7.4 ^a^	
Rarely–Never	72.1	71.3 ^a^	60.8 ^b^		94.9 ^a^	77.8 ^b^	
INSTITUTIONAL							
Location of food outlets on campus				<0.001			<0.001
Very Often–Often	11.9	8.4 ^a^	19.6 ^b^		4.0 ^a^	33.3 ^b^	
Sometimes	19.4	17.0 ^a^	22.8 ^a^		24.2 ^a^	11.1 ^a^	
Rarely–Never	68.7	74.6 ^a^	57.7 ^b^		71.7 ^a^	55.6 ^a^	
Campus food outlet hours				0.001			n.s.
Very Often–Often	13.9	11.0 ^a^	20.9 ^b^		10.1	18.5	
Sometimes	20.5	18.8 ^a^	23.0 ^a^		23.2	14.8	
Rarely–Never	65.6	70.2 ^a^	56.1 ^b^		66.7	66.7	
COMMUNITY							
Cost of food				<0.001			<0.001
Very Often–Often	23.2	11.3 ^a^	54.0 ^b^		4.1 ^a^	33.3 ^b^	
Sometimes	24.7	22.0 ^a^	33.3 ^b^		12.2 ^a^	44.4 ^b^	
Rarely–Never	52.2	66.8 ^a^	12.7 ^b^		83.7 ^a^	22.2 ^b^	
Lack of availability of cultural foods				0.002			n.s.
Very Often–Often	11.3	5.3 ^a^	15.9 ^b^		19.2	29.9	
Sometimes	11.7	8.9 ^a^	10.6 ^a^		20.2	25.9	
Rarely–Never	77.0	85.8 ^a^	73.5 ^b^		60.6	44.4	
Lack of reliable transportation				0.001			0.005
Very Often–Often	13.1	10.1 ^a^	19.6 ^b^		7.1 ^a^	29.6 ^b^	
Sometimes	12.6	10.6 ^a^	15.9 ^a^		13.1 ^a^	14.8 ^a^	
Rarely–Never	74.3	79.3 ^a^	64.6 ^b^		79.8 ^a^	55.6 ^b^	

^1^ Statistical test used was chi-square analysis for independence; n.s. = non-significant. Same superscript letters indicate column proportions that are not significantly different from each other.

**Table 3:** Food security coping strategies for the past 12 months by midwest university students by academic and food security status.

		Undergraduate (*n* = 549)			Graduate (*n* = 126)		
Food Security Coping Strategies	Total (%)	Food Secure 66% (360)	Food Insecure 34% (189)	*p*	Food Secure 79% (99)	Food Insecure 21% (27)	*p*
INTRAPERSONAL							
Bought cheapest food available even though knew it was not the healthiest.				<0.001			<0.001
Every month	19.1	12.0 ^a^	39.4 ^b^		6.1 ^a^	18.5 ^b^	
Some months during the year	29.5	24.6 ^a^	41.0 ^b^		19.2 ^a^	51.9 ^b^	
1 or 2 times in the year	17.7	19.3 ^a^	12.2 ^b^		21.2 ^a^	22.2 ^b^	
Never	33.7	44.1 ^a^	7.4 ^b^		53.5 ^a^	7.4 ^b^	
INTERPERSONAL							
Asked family or friends for help so had enough money to cover food costs.				<0.001			0.001
Every month	6.8	5.8 ^a^	11.6 ^b^		3.1 ^a^	0 ^a^	
Some months during the year	15.2	8.4 ^a^	36.0 ^b^		2.0 ^a^	7.4 ^a^	
1 or 2 times in the year	13.1	10.0 ^a^	17.5 ^b^		9.2 ^a^	37.0 ^b^	
Never	64.9	75.8 ^a^	34.9 ^b^		85.7 ^a^	55.6 ^b^	
Went hungry in order to use food money to go out somewhere social with friends.				<0.001			0.005
Every month	3.4	1.7 ^a^	6.9 ^b^		2.0 ^a^	7.4 ^a^	
Some months during the year	10.4	3.9 ^a^	25.4 ^b^		4.0 ^a^	14.8 ^b^	
1 or 2 times in the year	13.6	9.7 ^a^	24.9 ^b^		5.1 ^a^	18.5 ^b^	
Never	72.6	84.7 ^a^	42.9 ^b^		88.9 ^a^	59.3 ^b^	
Had to choose between paying for food or paying for housing or utilities.				<0.001			<0.001
Every month	2.2	0.3 ^a^	6.3 ^b^		0 ^a^	7.4 ^b^	
Some months during the year	5.8	0.5 ^a^	15.9 ^b^		1.0 ^a^	22.2 ^b^	
1 or 2 times in the year	7.3	1.9 ^a^	18.5 ^b^		4.0 ^a^	11.1 ^b^	
Never	84.7	97.3 ^a^	59.3 ^b^		94.9 ^a^	59.3 ^b^	
INSTITUTIONAL							
Had to choose between paying for food or paying for tuition, or other education expenses.				<0.001			<0.001
Every month	4.5	0.8 ^a^	12.8 ^b^		1.0 ^a^	7.4 ^b^	
Some months during the year	6.5	1.1 ^a^	16.6 ^b^		3.0 ^a^	22.2 ^b^	
1 or 2 times in the year	7.1	2.5 ^a^	17.1 ^b^		4.0 ^a^	11.1 ^a^	
Never	81.9	95.6 ^a^	53.5 ^b^		91.9 ^a^	59.3 ^b^	
COMMUNITY/POLICY							
Had to choose between paying for food or paying for medicine or medical care.				<0.001			<0.001
Every month	1.6	0.3 ^a^	5.3 ^b^		0	0	
Some months during the year	4.9	0.3 ^a^	13.2 ^b^		1.0 ^a^	22.2 ^b^	
1 or 2 times in the year	3.7	1.4 ^a^	9.0 ^b^		2.0 ^a^	3.7 ^a^	
Never	89.7	98.1 ^a^	72.5 ^b^		96.9 ^a^	74.1 ^b^	
Food pantry use				<0.001			0.001
Very Often-Sometimes	6.5	2.5 ^a^	10.1 ^b^		7.1 ^a^	33.3 ^b^	
Rarely–Never	93.5	97.5 ^a^	89.9 ^b^		92.9 ^a^	66.7 ^b^	

n.s. = non-significant. Same superscript letters indicate column proportions that are not significantly different from each other.

**Table 4:** Resource and food assistance program usage by midwest university students by academic and food security status ^1^.

		Undergraduate (*n* = 549)			Graduate (*n* = 126)		
Resource and Program Usage	Total (%)	Food Secure 66% (360)	Food Insecure 34% (189)	*p*	Food Secure 79% (99)	Food Insecure 21% (27)	*p*
INTRAPERSONAL							
How to cook simple, cheap, healthy meals				0.001			n.s.
Received and used	19.4	17.2 ^a^	22.6 ^a^		20.4	22.2	
Received but did not need	9.3	10.4 ^a^	9.7 ^a^		4.1	11.1	
Not received but would like	38.0	33.8 ^a^	45.2 ^b^		36.7	48.1	
Not received and do not need	33.3	38.6 ^a^	22.6 ^b^		38.8	18.5	
How to manage budget monthly living and college costs				<0.001			0.013
Received and used	18.4	18.5 ^a^	18.6 ^a^		17.3 ^a^	18.5 ^a^	
Received but did not need	12.2	12.6 ^a^	13.3 ^a^		7.1 ^a^	18.5 ^a^	
Not received but would like	32.2	25.5 ^a^	44.7 ^b^		28.6 ^a^	48.1 ^a^	
Not received and do not need	37.2	43.^a^	23.4 ^b^		46.9 ^a^	14.8 ^b^	
INSTITUTIONAL							
Campus resources if having trouble getting enough food				<0.001			0.014
Received and used	3.9	1.4 ^a^	9.0 ^b^		1.0 ^a^	11.1 ^b^	
Received but did not need	9.3	11.8 ^a^	5.9 ^b^		7.1 ^a^	7.4 ^a^	
Not received but would like	14.9	5.3 ^a^	26.1 ^b^		22.4 ^a^	37.0 ^a^	
Not received and do not need	71.9	81.5 ^a^	59.0 ^b^		69.4 ^a^	44.4 ^b^	
COMMUNITY							
Location of local food pantries, food banks, or free food sources				<0.001			0.001
Received and used	3.9	1.4 ^a^	7.4 ^b^		2.0 ^a^	18.5 ^b^	
Received but did not need	11.7	13.5 ^a^	10.1 ^a^		10.2 ^a^	3.7 ^a^	
Not received but would like	13.8	4.5 ^a^	22.3 ^b^		23.5 ^a^	40.7 ^a^	
Not received and do not need	70.7	80.6 ^a^	60.1 ^b^		64.3 ^a^	37.0 ^b^	
POLICY							
How to apply for federal food assistance programs (e.g., SNAP ^2^)				<0.001			0.042
Received and used	3.3	1.7 ^a^	5.9 ^b^		3.1 ^a^	7.4 ^a^	
Received but did not need	4.9	3.7 ^a^	8.0 ^b^		3.1 ^a^	7.4 ^a^	
Not received but would like	11.1	3.1 ^a^	19.1 ^b^		17.3 ^a^	37.0 ^b^	
Not received and do not need	80.7	91.5 ^a^	67.0 ^b^		76.5 ^a^	48.1 ^b^	

^1^ Statistical test used was chi-square analysis for independence; n.s. = non-significant. Same superscript letters indicate column proportions that are not significantly different from each other. ^2^ Supplemental Nutrition Assistance Program.

**Table 5:** Logistic regression model of predictors of food insecurity among midwest university undergraduate and graduate students.

			**95% Confidence Interval for Odds Ratio**		
Undergraduate Model	**Beta (SE ^1^)**	***p***	**Lower**	**Odds Ratio**	**Upper**
INTRAPERSONAL					
Lower view of health status	0.606 (0.146)	<0.001	1.377	1.834	2.442
Receive financial aid (1)	1.047 (0.254)	<0.001	1.732	2.848	4.685
Non-White race (1)	0.512 (0.323)	0.113	0.886	1.669	3.146
INTERPERSONAL					
Housing—off campus (1)	0.891 (0.280)	0.001	1.408	2.436	4.216
INSTITUTIONAL					
Campus food store hours	0.243 (0.106)	0.021	1.037	1.275	1.569
Employment—working (1)	0.539 (0.272)	0.047	1.007	1.715	2.921
COMMUNITY					
Cost of food as barrier	1.100 (0.113)	<0.001	2.410	3.006	3.749
Constant	−7.698 (0.737)	<0.001		<0.001	
			**95% Confidence Interval for Odds Ratio**		
Graduate Model	**Beta (SE ^1^)**	***p***	**Lower**	**Odds Ratio**	**Upper**
INTRAPERSONAL					
Asian ethnicity (1)	1.744 (0.750)	0.020	1.315	5.720	24.875
No time to prepare food	0.663 (0.339)	0.050	0.999	1.941	3.771
Lack of foods for diet needs	−0.725 (0.380)	0.056	0.230	0.484	1.020
INSTITUTIONAL					
Employment—working (1)	−1.471 (0.874)	0.092	0.041	0.230	1.274
COMMUNITY					
Cost of food as barrier	2.061 (0.434)	<0.001	3.352	7.851	18.387
Constant	−6.717 (1.751)	<0.001		0.001	

^1^ Standard Error.

## Data Availability

The data presented in this study are available on request from the corresponding author. The data are not publicly available due to privacy reasons.
